# A Retrospective Database Analysis to Estimate the Burden of Acute Otitis Media in Children Aged <15 Years in the Veneto Region (Italy)

**DOI:** 10.3390/children9030436

**Published:** 2022-03-19

**Authors:** Elisa Barbieri, Gloria Porcu, Tianyan Hu, Tanaz Petigara, Francesca Senese, Gian Marco Prandi, Antonio Scamarcia, Luigi Cantarutti, Anna Cantarutti, Carlo Giaquinto

**Affiliations:** 1 Department of Women’s and Children’s Health, Division of Paediatric Infectious Diseases, University of Padova, 35128 Padova, Italy; carlo.giaquinto@unipd.it; 2 Unit of Biostatistics Epidemiology and Public Health, Department of Statistics and Quantitative Methods, University of Milano-Bicocca, 20126 Milan, Italy; gloria.porcu@unimib.it (G.P.); anna.cantarutti@unimib.it (A.C.); 3 National Centre for Healthcare Research and Pharmacoepidemiology, Department of Statistics and Quantitative Methods, University of Milano-Bicocca, 20216 Milan, Italy; 4 Center for Observational and Real-World Evidence, Merck & Co., Inc., Kenilworth, NJ 07033, USA; tianyan.hu@merck.com (T.H.); tanaz.petigara@merck.com (T.P.); 5 MSD Italy, Via Vitorchiano 151, 00189 Rome, Italy; francesca.senese@merck.com (F.S.); gian.marco.prandi@merck.com (G.M.P.); 6 Pedianet Project, 35100 Padova, Italy; a.scamarcia@sosepe.com (A.S.); l.cantarutti@sosepe.com (L.C.)

**Keywords:** acute otitis media, recurrent acute otitis media, incidence, pneumococcal vaccination

## Abstract

This study aimed to assess trends in the incidence of acute otitis media (AOM), a common childhood condition, following the introduction of the 13-valent pneumococcal conjugate vaccine (PCV13) in the Veneto region of Italy in 2010. AOM episodes (overall, simple, and recurrent (≥3 or ≥4 episodes in 6 or 12 months, respectively, with ≥1 episode in the preceding 6 months)) in children <15 years of age were identified in Pedianet from 2010–2017. Interrupted time series analyses were conducted to assess changes in the annual incidence rates (IRs) in early (2010–2013) and late (2014–2017) PCV13 periods. In total, 72,570 children (402,868 person-years) were identified; 21,048 had 41,683 AOM episodes. Mean annual AOM IR was 103/1000 person-years (95% confidence interval: 102–104), decreasing from 126 to 79/1000 person-years. AOM IRs were highest in children 2–4 years of age, followed by <2 and 5–14 years of age. Overall and simple AOM IRs decreased among children 0–14 years of age, including 2–4 and 5–14 years of age, while recurrent AOM IRs decreased in children <2 years of age. Following PCV13 introduction, AOM IRs decreased substantially in children <15 years of age, with the greatest benefit observed in older children, driven by a reduction in simple AOM IRs. AOM disease burden remains substantial.

## 1. Introduction

Acute otitis media (AOM) is one of the most common infectious diseases in young children [1]. By 3 years of age, 60–80% of children have had at least 1 episode of AOM [2,3], with 40% of children having had at least 6 episodes by 7 years of age [4]. Further, 3 bacterial pathogens, *Streptococcus pneumoniae*, non-typeable *Haemophilus influenzae* (NTHi), and *Moraxella catarrhalis*, account for almost 90% of non-recurrent AOM infections, with 25–44% attributable to *S. pneumoniae* [3]. Although the majority of cases of AOM can be attributed to bacterial and viral co-infection, around 10% of AOM cases are solely associated with viral pathogens [5]. These include respiratory syncytial virus (RSV), influenza virus, adenovirus, and parainfluenza [6]. The risk of AOM can be linked to a multitude of factors. Environmental aspects, such as low socioeconomic status, attending day care, and exposure to smoking, can all contribute to poorer living conditions, increasing the risk of otitis media [7]. Regarding the host, genetic factors, such as polymorphisms that regulate the immune response, or a family history of otitis media can increase the risk of AOM occurrence. An individual who is of younger age and male sex is more likely to be diagnosed with AOM [7]. Similarly, changes in eustachian tube anatomy, specifically the enlargement of the adenoids and nasopharynx, can increase the risk of bacterial or viral infection, resulting in obstruction of the eustachian tubes and an increased risk of AOM [7,8]. Recurrent infections linked to *S. pneumoniae* are common and can lead to post-infectious complications, such as perforated tympanic membrane [9,10,11,12].

Vaccination for the prevention of bacterial infections, such as those caused by *H. influenzae* and *S. pneumoniae,* remains the most important and effective public health tool to reduce the burden of AOM.

The Veneto region is the fifth-largest region in Italy, with just over 4.9 million inhabitants, including approximately 700,000 children less than 15 years of age. The 7-valent pneumococcal conjugate vaccine (PCV7) was introduced into the Veneto regional immunization program in 2003 and was replaced with the 13-valent PCV (PCV13) in 2010 [13].

Various studies in Europe and elsewhere have suggested a decline in the incidence of AOM following the introduction of PCVs [14,15,16,17,18,19,20,21,22,23]. In southern Italy, specifically, 1 study conducted in the Puglia region between 2006 and 2012 showed a reduction of almost 40% in hospitalization rates linked to AOM complications in children <5 years of age following the introduction of PCV7 [24]. Following the introduction of PCV13 into the Italian immunization program in 2010, a high uptake across the country has been reported, with between 76% and 96% of infants vaccinated in 2018 [25,26]. However, there is a lack of current evidence on the clinical burden of AOM in Italy.

New vaccines are in development to further reduce the burden of pneumococcal disease, including AOM. The investigational vaccines contain all serotypes in the currently licensed PCV13 and additional serotypes [27,28]. To better understand the current burden of AOM and the potential value of new vaccines in the Veneto region of Italy, it is important to quantify the incidence and trends of AOM following the introduction of PCV13, and the residual burden of AOM, prior to the introduction of new vaccines. In this study, the incidence rate (IR) and time trends for overall AOM, and for simple and recurrent AOM separately, were estimated in a primary care setting after the introduction of PCV13 in the Veneto region of Italy.

## 2. Materials and Methods

### 2.1. Study Design, Population, and Case Identification

This retrospective observational analysis included all children who resided in the Veneto region and were <15 years of age between 1 January 2010 and 31 December 2017. AOM episodes were identified in children registered in the Pedianet database for at least 6 months prior to their first AOM episode (i.e., the index episode) and for at least 12 months after the index date; the 6-month pre-index window was not required for children <1 year of age.

AOM diagnoses were identified using the International Classification of Diseases, Ninth Revision, Clinical Modification system (ICD-9-CM; codes: 382.x). Any visits with these ICD-9-CM codes in primary or secondary positions were identified as AOM-related visits. Diagnoses were also identified using free text algorithms (“oti*”, “*oti*”, “om*”, and “*om*”), as validated in other studies [20,29], and were manually selected and categorized according to pneumococcal etiology (where specified) and confirmed by a clinical expert to exclude false positives.

An AOM episode comprised 1 or more primary care AOM-related visits; if additional primary care visits with the same diagnosis within 14 days of the first visit were found for any patient, they were considered follow-up visits. Thus, a gap of more than 14 days between AOM visits defined the start of a new episode. Episodes that crossed calendar years were assigned to the year in which the episode began. AOM episodes were further classified as recurrent AOM (at least 3 episodes within 6 months or at least 4 episodes within 12 months, with at least 1 episode in the preceding 6 months [30]) and simple AOM (AOM episodes not classified as recurrent AOM).

AOM episodes in children for whom age data were missing in any calendar year during the study period were excluded from the analyses, as were children with fewer than 2 distinct visits.

### 2.2. Data Sources

Data were retrieved from Pedianet (http://www.pedianet.it) (accessed on 1 June 2021), a pediatric primary care database in which data from an established Italian network of 130 family physicians are collated, as previously described [31,32]. Data generated during routine patient care using a common software (JuniorBit^®^) are anonymized and sent monthly to a centralized database in Padova for validation. The database includes patient demographics and clinical characteristics, including diagnoses (free text or ICD-9-CM codes), symptoms, and other medical observations related to the visits, ambulatory diagnostic exams, pharmaceutical prescriptions (identified by Anatomical Therapeutical Chemical codes), specialist visits, and diagnostic procedures. Inclusion in the Pedianet database is voluntary. Parents/legal guardians provided consent for their children’s data to be used for research purposes.

There is also a centralized database for all prescriptions, specialist visits, emergency room (ER) visits, and hospitalizations in the Veneto region. This database is populated by different local health authorities and hospital trusts for administrative and reimbursement purposes. Data for hospitalization and ER visits (including anonymized discharge letters) are individually linked to the Pedianet database through the Fascicolo Sanitario Project for all children for whom informed consent for the linkage was provided.

This study’s design and access to the Pedianet database were approved by the Internal Scientific Committee of So.Se.Pe. Srl, the legal owner of Pedianet.

### 2.3. Statistical Analysis

The demographic characteristics and risk factors for AOM included in this analysis are described for all children <15 years of age, and separately for children <2 years, 2–4 years, and 5–14 years of age. Demographic characteristics include sex. Risk factors for AOM include a history of pre-term birth (<37 gestational weeks) and the presence of 1 or more medical conditions, such as diabetes, chronic heart disease, and chronic lung disease, which are listed in full in the Supplementary Materials.

Mean annual IRs and corresponding 95% confidence intervals (CIs) were estimated and are reported as the number of episodes per 1000 person-years. Person-years accumulated from the date when the patient was registered in the Pedianet database until death, migration, or end of follow-up (the date at which the physician ended care (date of last follow-up), or 31 December 2017, whichever occurred first). The following formula was used to calculate the 95% CIs: ($IR\;\pm 1.96*\sqrt{\frac{events}{person-years^{2}\;}}*1000)$.

IRs were also calculated by age group (<2 years, 2–4 years, and 5–14 years of age) and sex (male/female).

Interrupted time series (ITS) analyses were conducted to estimate the differential trends in the annual IRs in the early (2010–2013) and late (2014–2017) PCV13 periods, using a negative binomial regression model. For each period, changes in levels (i.e., immediate changes in the IRs compared with the previous period), and changes in trend (i.e., gradual changes in the IRs over time compared with trends in the previous period) were assessed. Models were adjusted for the demographic characteristics and risk factors described above. ITS analysis was performed for all children <15 years of age and then separately for each of the 3 age groups. The Mann–Kendall (MK) trend test was used to estimate whether the trend in AOM incidence was monotonic during 2010–2017 [33].

The analyses described above were repeated for recurrent and simple AOM episodes. All analyses were performed using the Statistical Analysis System software (version 9.4; SAS Institute, Cary, NC, USA). Statistical significance was set at *p =* 0.05.

## 3. Results

In total, 72,570 children <15 years of age (402,868 person-years) between 2010 and 2017 were identified in the Pedianet database. Of these, 21,047 children experienced 41,683 AOM episodes (22,245 (53%) were in males), and 1971 (5%) were in children born before 37 gestational weeks. The most common medical conditions were chronic heart disease (present in 159 AOM episodes, 0.4%) and asthma (present in 132 AOM episodes, 0.3%).

The mean annual IR of AOM in children <15 years of age was 103 (95% CI: 102–104) per 1000 person-years during the study period. The IR decreased from 126 in 2010 to 79 per 1000 person-years in 2017 (Table 1). From 2010–2017, IRs for AOM decreased in children 2–4 years of age (from 213 to 178 per 1000 person-years, MK test: *p =* 0.0065) and in children 5–14 years of age (from 60 to 38 per 1000 person-years, *p* < 0.001), and increased from 179 to 186 per 1000 person-years in children <2 years of age (*p =* 0.0478) (Table 1).

The mean annual IRs of AOM by sex and age group are shown in Table 2. IRs of AOM were slightly higher in males than in females (107 and 100 per 1000 person-years, respectively).

**Figure 1:**
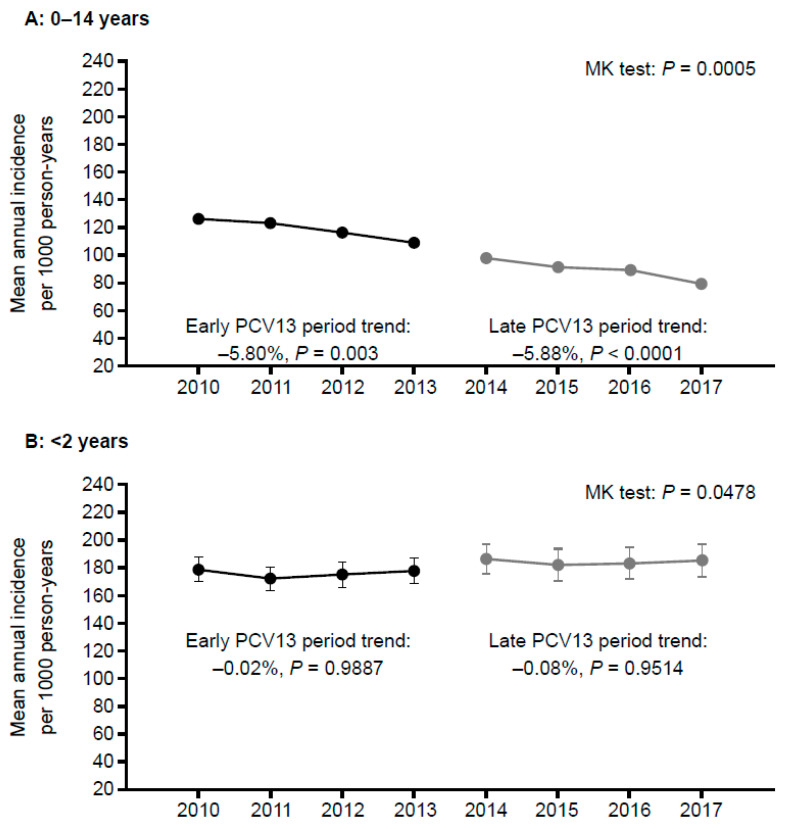

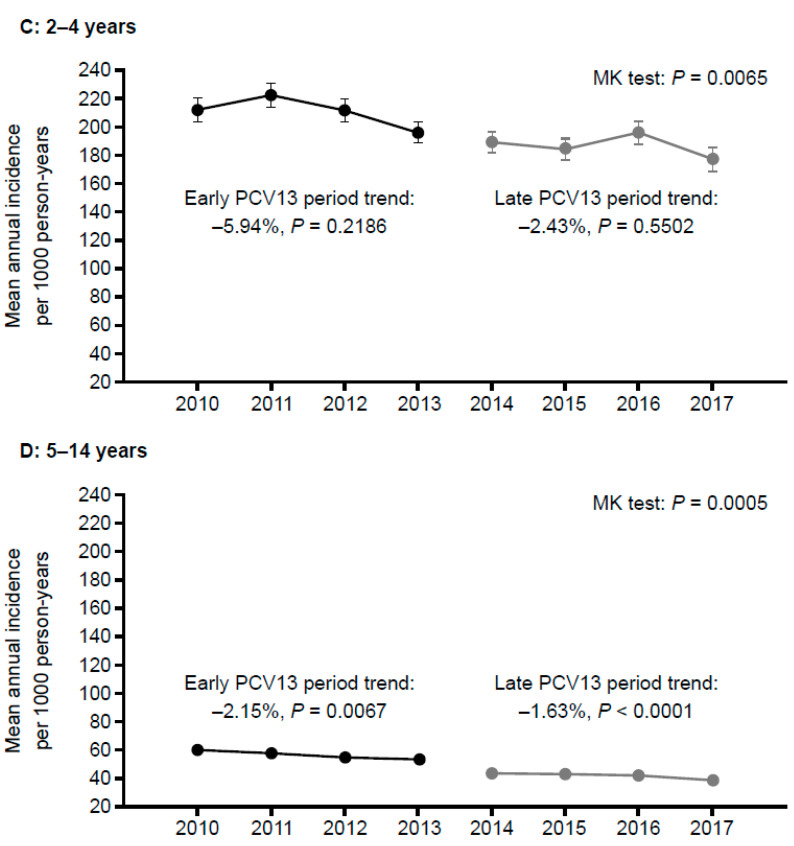
Interrupted time series of mean annual AOM incidence in the pediatric population: (**A**) 0–14 years; (**B**) <2 years; (**C**) 2–4 years; and (**D**) 5–14 years of age; AOM, acute otitis media; MK, Mann–Kendall; PCV13, 13-valent pneumococcal conjugate vaccine.

The results of the ITS analysis are displayed in Figure 1. Among children <15 years of age, the IRs of AOM decreased gradually, by 6% per year, in the early (*p =* 0.003) and late (*p* < 0.001) PCV13 period. There was no significant immediate change in the IRs between the early and late PCV13 periods (Figure 1A).

The ITS analysis also showed no significant gradual or immediate changes in IRs of AOM in both the early and late PCV13 periods for children <2 and 2–4 years of age (Figure 1B,C). However, in children 5–14 years of age, IRs decreased gradually, by 2% per year, in the early (*p =* 0.007) and late (*p* < 0.001) PCV13 period (Figure 1D). There was also an immediate reduction of 8% between the IRs in the early and late PCV13 periods (*p =* 0.005).

### Simple and Recurrent AOM

In all children <15 years of age, the annual IRs of simple AOM decreased from 110 to 69 per 1000 person-years between 2010 and 2017 (MK test: *p* < 0.001) (Supplementary Table S1 and Figure S1A). The decrease was mostly driven by reductions in children 2–4 years of age (from 187 to 152 per 1000 person-years, MK test: *p =* 0.026) and 5–14 years of age (from 58 to 33 per 1000 person-years, MK test: *p =* 0.0013). By contrast, the IRs for simple AOM in children <2 years of age increased from 140 to 168 per 1000 person-years between 2010 and 2017 (MK test: *p =* 0.0065). The results of ITS analysis (Supplementary Figure S1) confirmed the statistical significance of these changes.

For recurrent AOM, the IRs decreased from 16 to 11 per 1000 person-years in children <15 years of age during this time, with no significant trend detected (MK test: *p =* 0.083) (Supplementary Table S2 and Figure S2A). The IRs decreased only in children <2 years of age (from 39 to 18 per 1000 person-years, MK test: *p =* 0.003) (Supplementary Figure S2).

## 4. Discussion

The results of this study showed that the IRs of AOM declined significantly between 2010 and 2017 in the overall cohort of children <15 years of age from Veneto, Italy. The IR of AOM increased over the study period in children <2 years of age and decreased in those 2–4 and 5–14 years of age. Results from this study also showed that IRs of both simple and recurrent AOM decreased from 2010–2017 in all children <15 years of age. The IRs of simple AOM decreased in children 2–4 and 5–14 years of age while the IRs of recurrent AOM decreased only in children <2 years of age.

The reduction in AOM episodes since the introduction of PCVs is consistent with previous studies on AOM burden in Europe [14,20,21,22,23,34]. The introduction of PCV7 and PCV13 in the United Kingdom was associated with significant reductions (22% and 19%, respectively) in AOM cases in children <10 years of age between 2002 and 2012 [13]. In Sweden, outpatient specialist AOM visits in children 0–4 years of age decreased from 47 to 39 per 1000 person-years between 2005 and 2014 [34]. In a study in Germany, Italy, Spain, Sweden, and the United Kingdom in children <6 years of age, the incidence of AOM gradually decreased from 268 per 1000 person-years in 2007–2008 to 256 per 1000 person-years in 2008–2010 [20]. Similarly, the incidence of AOM in children <6 years of age decreased in 5 countries in Eastern Europe between 2011 and 2013, from 182 to 161 per 1000 person-years [21].

In our study, a reduction in overall AOM incidence was only observed in older children 5–14 years of age, with significant reductions in both the early and late PCV13 periods. No significant trends were observed in the <2 and 2–4 years age groups in either period. The lack of significant trends in the younger children could be indicative of the incidence of AOM plateauing following an initial reduction, as shown in the United Kingdom. In a study in the United Kingdom, IRs of AOM in children <2 and 2–4 years of age decreased significantly following the introduction of PCV7 but leveled off during the post-PCV13 period in both age groups [14]. In addition, in an earlier study using data from Pedianet, there was a 40% reduction in AOM hospitalizations in children <5 years of age following the introduction of PCV7 [29]. The leveling-off of AOM IRs in younger children could also be due to the impact of the PCV13 program on the etiology of AOM—any change in the proportions of attributable pathogens or the pneumococcal serotype distribution could affect the incidence of AOM. The increase in AOM incidence in children <2 years of age after 2013 could be attributed to the vaccination program, as PCV13 vaccination is not mandatory. Between 2013 and 2016 in Veneto, the regional coverage of the first dose of PCV13 decreased to below 90%, and completion of the vaccination schedule was achieved in 85–87% of the birth cohort.

In the current study, the incidence of overall AOM was higher in children 2–4 years of age than in those <2 years of age [20]. Similar results were obtained in a multicenter observational study conducted between 2011 and 2013 in Eastern Europe, in which the highest IR of AOM was 208.9 per 1000 person-years in children 3–4 years of age compared with 92.3 per 1000 person-years in children <1 year of age [21]. There are multiple possible explanations. It is well recognized that daycare attendance is associated with an increased risk of AOM [35,36]. The results of a study conducted in Italy [29] suggested that the higher average age of children attending pre-nursery or nursery schools in Italy and the decreasing average number of individuals in a family could explain the lower IRs for AOM in children <2 years of age compared with other European countries [14,20].

Despite the overall reduction in the IR of AOM in children <15 years of age, there is a residual burden of AOM in the Veneto region of Italy, with a mean annual IR of 103 per 1000 person-years. In addition to the use of PCVs with increasing serotype coverage for *S. pneumoniae,* co-administration with a vaccine that elicits antibodies that are cross-reactive to *H. influenzae* may be an alternative strategy to address this residual disease burden. As previously discussed, three bacterial pathogens, *S. pneumoniae*, NTHi, and *M. catarrhalis*, are responsible for the majority of non-recurrent AOM infections [3]. In a murine model of AOM, Rowe et al. studied the effectiveness of a pneumococcal surface protein C (PspC) vaccine that elicits antibodies that are cross-reactive against NTHi, co-administered with PCV13. Results demonstrated a significant reduction in AOM incidence and bacterial burden in the middle ear through the generation of cross-reactive antibodies recognizing multiple bacterial pathogens [37]. If successful in human studies, this strategy could considerably reduce the burden of AOM in children by broadening the protection against both pneumococcus and other AOM pathogens, such as NTHi.

To our knowledge, this is the first European study to investigate outpatient IRs of AOM in the PCV13 era. The strengths of our study include its size, generalizability, and representative coverage of pediatric patients; however, the study is limited by its retrospective nature. Parents may adopt a “watchful waiting” approach, which involves waiting for 48–72 h (administering medication to reduce pain, if needed) and then re-evaluating the necessity for antibiotics. As AOM can resolve spontaneously and there is no need for a prescription to obtain pain medication, some AOM episodes may not have been detected. Nevertheless, a previous European study in which data from a retrospective medical chart review were compared with those from a prospective survey of parents did not detect any significant differences in the IR of AOM between the two sources in Italian children [20]. Although it is possible that some cases of AOM were seen in an ER without being recorded in the database, it is likely that these cases would have been identified at a later stage, because a follow-up examination by a family pediatrician is often recommended after ER discharge, especially for younger children. Furthermore, data on hospitalization and ER admissions for patients who consented to participate in the Fascicolo Sanitario Project were directly linked to the Pedianet database. Neither the use of a pneumatic otoscope nor the results of tympanostomy exams were retrieved from the database; thus, no sensitivity analyses could be conducted to determine whether diagnoses were subjective to the attending clinicians (although this would be unlikely to affect the trends observed). Finally, other factors, such as breastfeeding, smokers, older siblings in the household, and daycare attendance, were not considered in this analysis.

## 5. Conclusions

After the introduction of PCV13 in the Veneto region of Italy, IR of AOM decreased significantly in children <15 years of age. Overall, the greatest benefits were observed in older children, driven by a reduction in IR for simple AOM. However, the disease burden of AOM remains substantial in the Veneto region of Italy. The impact of future PCVs on IR of AOM will depend on the proportion of AOM caused by *S. pneumoniae* and vaccine-type serotypes.

## Figures and Tables

**Table 1:** Annual IR of AOM in the overall pediatric population 0–14 years of age and by age group.

Year	Number of Episodes (*n* = 41,683)	Person-Years	Annual IR per 1000 Person-Years (95% CI)
**Overall** **(0** **–** **14 years of age)**			
2010	5510	43,692.02	126 (123–129)
2011	5871	47,609.77	123 (120–126)
2012	5895	50,643.46	116 (113–119)
2013	5694	52,192.36	109 (106–112)
2014	5195	52,805.74	98 (96–101)
2015	4783	52,392.12	91 (89–94)
2016	4658	52,184.01	89 (87–92)
2017	4077	51,348.38	79 (77–82)
**<2 years of age**			
2010	1504	8396.77	179 (170–188)
2011	1500	8687.03	173 (164–181)
2012	1483	8475.04	175 (166–184)
2013	1383	7757.59	178 (169–188)
2014	1259	6746.95	187 (176–197)
2015	998	5460.51	183 (171–194)
2016	953	5192.16	184 (172–195)
2017	960	5159.47	186 (174–198)
**2** **–4 years of age**			
2010	2625	12,338.81	213 (205–221)
2011	2861	12,846.22	223 (215–231)
2012	2816	13,289.06	212 (204–220)
2013	2641	13,436.86	197 (189–204)
2014	2503	13,207.4	190 (182–197)
2015	2294	12,405.18	185 (177–192)
2016	2189	11,145.09	196 (188–205)
2017	1710	9629.78	178 (169–186)
**5** **–14 years of age**			
2010	1381	22,956.44	60 (57–63)
2011	1510	26,076.52	58 (55–61)
2012	1596	28,879.35	55 (53–58)
2013	1670	30,997.91	54 (51–56)
2014	1433	32,851.39	44 (41–46)
2015	1491	34,526.43	43 (41–45)
2016	1516	35,846.76	42 (40–44)
2017	1407	36,559.13	38 (36–40)

AOM: acute otitis media, CI: confidence interval, IR: incidence rate.

**Table 2:** Mean annual IRs of AOM by sex and age group.

	Number of Episodes (*n* = 41,683)	Person-Years	Annual IR per 1000 Person-Years (95% CI)
**Sex**			
Male	22,245	208,832.96	107 (105–108)
Female	19,438	194,034.89	100 (99–102)
**Age**			
<2 years	10,040	55,875.52	180 (176–183)
2–4 years	19,639	98,298.40	200 (197–203)
5–14 years	12,004	248,693.93	48 (47–49)

AOM: acute otitis media, CI: confidence interval, IR: incidence rate.

## Data Availability

The data used in this study cannot be made available in the manuscript, the Supplementary Materials, or in a public repository due to Italian data protection laws. The anonymized datasets generated during and/or analyzed during the current study can be provided on reasonable request, from the corresponding author, after written approval by the Internal Scientific Committee (info@pedianet.it).

## Supplementary Materials

The following supporting information can be downloaded at: https://www.mdpi.com/article/10.3390/children9030436/s1, Figure S1: Interrupted time series of mean annual simple AOM incidence in the pediatric population: (A) 0–14 years; (B) <2 years; (C) 2–4 years; and (D) 5–14 years of age; Figure S2: Interrupted time series of mean annual recurrent AOM incidence in the pediatric population: (A) 0–14 years; (B) <2 years; (C) 2–4 years; and (D) 5–14 years of age; Table S1: Annual IR of simple AOM in the overall pediatric population 0–14 years of age and by age group; Table S2: Annual IRs of recurrent AOM in the overall pediatric population 0–14 years of age and by age group.
