# Supplementary material for: A Retrospective Database Analysis to Estimate the Burden of Acute Otitis Media in Children Aged <15 Years in the Veneto Region (Italy)
Source: Children (Basel). 2022 Mar 19;9(3):436. doi: 10.3390/children9030436 (PMC8947099; doi:10.3390/children9030436)
Supplement: Supplementary file 1 [file children-09-00436-s001.zip › children-1524614-supplementary.pdf]

Supplement

For the statistical analysis, the following medical conditions were considered to be risk factors for AOM in the study

Diabetes mellitus, chronic heart disease, chronic lung disease, asthma, chronic liver disease, cochlear implant, cerebrospinal fluid leak, sickle cell disease and other hemoglobinopathies, anatomic or functional asplenia, congenital or acquired immunodeficiency, infection with human immunodeficiency virus, chronic renal failure, nephrotic syndrome, malignant neoplasms, leukemias, lymphomas, Hodgkin’s disease, and other diseases associated with treatment with immunosuppressive drugs or radiation therapy, solid organ transplantation, or multiple myeloma.

We thank the following physicians for their contribution of data through participation in Pedianet:

Angelo Alongi, Roberta Angelini, Giovanni Avarello, Lucia Azzoni, Maria Carolina Barbazza, Maria Barberi Frandanisa, Patrizia Barbieri, Gabriele Belluzzi, Eleonora Benetti, Roberto Bezzi, Franca Boe, Stefano Bollettini, Andrea Bruna, Ivana Brusaterra, Roberto Budassi, Massimo Caccini, Laura Cantalupi, Luigia Caprio, Massimo Castaldo, Stefano Castelli, Serenella Castronuovo, Monica Cavedagni, Stefania Censini, Giuseppe Egidio Cera, Carla Ciscato, Mariangela Clerici Schoeller, Giuseppe Collacciani, Fabrizio Comaita, Ugo Alfredo Conte, Nicola Costanzo, Sandra Cozzani, Giancarlo Cuboni, Vito Francesco D’Amanti, Rita De Angelis, Roberto De Clara, Annamaria De Marchi, Emanuele De Nicolò, Gian Piero Del Bono, Gigliola Del Ponte, Tiziana Di Giampietro, Giuseppe Di Mauro, Giuseppe Di Santo, Piero Di Saverio, Marco Dolci, Mattia Doria, Stefano Drago, Pietro Falco, Mario Fama, Marco Faraci, Tania Favilli, Mariagrazia Federico, Michele Felice, Enrico Ferrara, Marta Ferrarese, Michele Ferretti, Paolo Forcina, Claudio Frattini, Ezio

Frison, Fabrizio Fusco, Giovanni Gallo, Andrea Galvagno, Alberta Gentili, Pierfrancesco Gentilucci, Giuliana Giampaolo, Giuseppe Giancola, Silvia Girotto, Costantino Gobbi, Mauro Grelloni, Mirco Grugnetti, Urania Lagrasta, Massimo Landi, Paola Lasalvia, M. Rosaria Letta, Giuseppe Lietti, Cinzia Lista, Ricciardo Lucantonio, Francesco Luise, Diego Luotti, Nadia Macropodio, Francesca Marine, Lorenzo Mariniello, Gabriele Marostica, Sergio Masotti, Stefano Meneghetti, Massimo Milani, Stella Vittoria Milone, Angela Maria Monteleone, Pierangela Mussinu, Carmen Muzzolini, Flavia Nicoloso, Laura Olimpi, Maria Maddalena Palma, Vittorio Pandolfini, Angela Pasinato, Andrea Passarella, Pasquale Pazzola, Monica Perin, Danilo Perri, Silvana Pescosolido, Giovanni Petrazzuoli, Giuseppe Petrotto, Patrizia Picco, Ambrogina Pirola, Lorena Pisanello, Daniele Pittarello, Eleonora Polidoro, Elena Porro, Adolfo Francesco Porto, Elisabetta Profumo, Antonino Puma, Ferdinando Ragazzon, Paolo Rosas, Rino Rosignoli, Mariagiulia Rosina, Mariella Rossitto, Bruno Ruffato, Lucia Ruggieri, Annamaria Ruscitti, Annarita Russo, Pietro Salamone, Daniela Sambugaro, Luigi Saretta, Vittoria Sarno, Nico Maria Sciolla, Paolo Senesi, Carla Silvan, Valter Spanevello, Francesco Speranza, Maura Sticco, Francesco Storelli, Gianni Tamassia, Paolo Tambaro, Giacomo Toffol, Marco Tondello, Gabriele Tonelli, Angelo Tummarello, Sergio Venditti, Concetta Volpe, Francescopaolo Volpe, Aldo Vozzi.

Supplementary Table S1. Annual IR of simple AOM in the overall pediatric population 0–14 years of age and by age group

Year	Simple AOM episodes (n = 34,739)	Person-years	Annual IR per 1000 person-years (95% CI)
**Overall (0–14 years of age)**			
2010	4809	43,692.02	110 (107–113)
2011	4957	47,609.77	104 (101–107)
2012	4769	50,643.46	94 (91–97)
2013	4683	52,192.36	90 (87–92)
2014	4191	52,805.74	79 (77–82)
2015	3923	52,392.12	75 (73–77)
2016	3871	52,184.01	74 (72–77)
2017	3536	51,348.38	69 (67–71)
**<2 years of age**			
2010	1177	8396.77	140 (132–148)
2011	1173	8687.03	135 (127–143)
2012	1148	8475.04	135 (128–143)
2013	1096	7757.59	141 (133–150)
2014	1034	6746.95	153 (144–163)
2015	827	5460.51	151 (141–162)
2016	810	5192.16	156 (145–167)
2017	867	5159.47	168 (157–179)
**2–4 years of age**			
2010	2308	12,338.81	187 (179–195)
2011	2361	12,846.22	184 (176–191)
2012	2164	13,289.06	163 (156–170)
2013	2083	13,436.86	155 (148–162)
2014	1929	13,207.40	146 (140–153)
2015	1801	12,405.18	145 (138–152)

2016	1768	11,145.09	159 (151–166)
2017	1462	9629.78	152 (144–160)
**5–14 years of age**			
2010	1324	22,956.44	58 (55–61)
2011	1423	26,076.52	55 (52–57)
2012	1457	28,879.35	50 (48–53)
2013	1504	30,997.91	49 (46–51)
2014	1228	32,851.39	37 (35–39)
2015	1295	34,526.43	38 (35–40)
2016	1293	35,846.76	36 (34–38)
2017	1207	36,559.13	33 (31–35)

Abbreviations: AOM, acute otitis media; CI, confidence interval; IR, incidence rate.

Supplementary Table S2. Annual IRs of recurrent AOM in the overall pediatric population 0–14 years of age and by age group

Year	Recurrent AOM episodes (n = 6944)	Person-years	Annual IR per 1000 person-years (95% CI)
**Overall (0–14 years of age)**			
2010	701	43,692.02	16 (15–17)
2011	914	47,609.77	19 (18–20)
2012	1126	50,643.46	22 (21–24)
2013	1011	52,192.36	19 (18–21)
2014	1004	52,805.74	19 (18–20)
2015	860	52,392.12	16 (15–18)
2016	787	52,184.01	15 (14–16)
2017	541	51,348.38	11 (10–11)
**<2 years of age**			
2010	327	8396.77	39 (35–43)
2011	327	8687.03	38 (34–42)
2012	335	8475.04	40 (35–44)
2013	287	7757.59	37 (33–41)
2014	225	6746.95	33 (29–38)
2015	171	5460.51	31 (27–36)
2016	143	5192.16	28 (23–32)
2017	93	5159.47	18 (14–22)
**2–4 years of age**			
2010	317	12,338.81	26 (23–29)
2011	500	12,846.22	39 (36–42)
2012	652	13,289.06	49 (45–53)
2013	558	13,436.86	42 (38–45)
2014	574	13,207.40	43 (40–47)
2015	493	12,405.18	40 (36–43)

2016	421	11,145.09	38 (34–41)
2017	248	9629.78	26 (23–29)
**5–14 years of age**			
2010	57	22,956.44	2 (2–3)
2011	87	26,076.52	3 (3–4)
2012	139	28,879.35	5 (4–6)
2013	166	30,997.91	5 (5–6)
2014	205	32,851.39	6 (5–7)
2015	196	34,526.43	6 (5–6)
2016	223	35,846.76	6 (5–7)
2017	200	36,559.13	5 (5–6)

Abbreviations: AOM, acute otitis media; CI, confidence interval; IR, incidence rate.

Supplementary Figure S1. Interrupted time series of mean annual simple AOM incidence in the pediatric population: A) 0–14 years; B) <2 years; C) 2–4 years; and D) 5–14 years of age

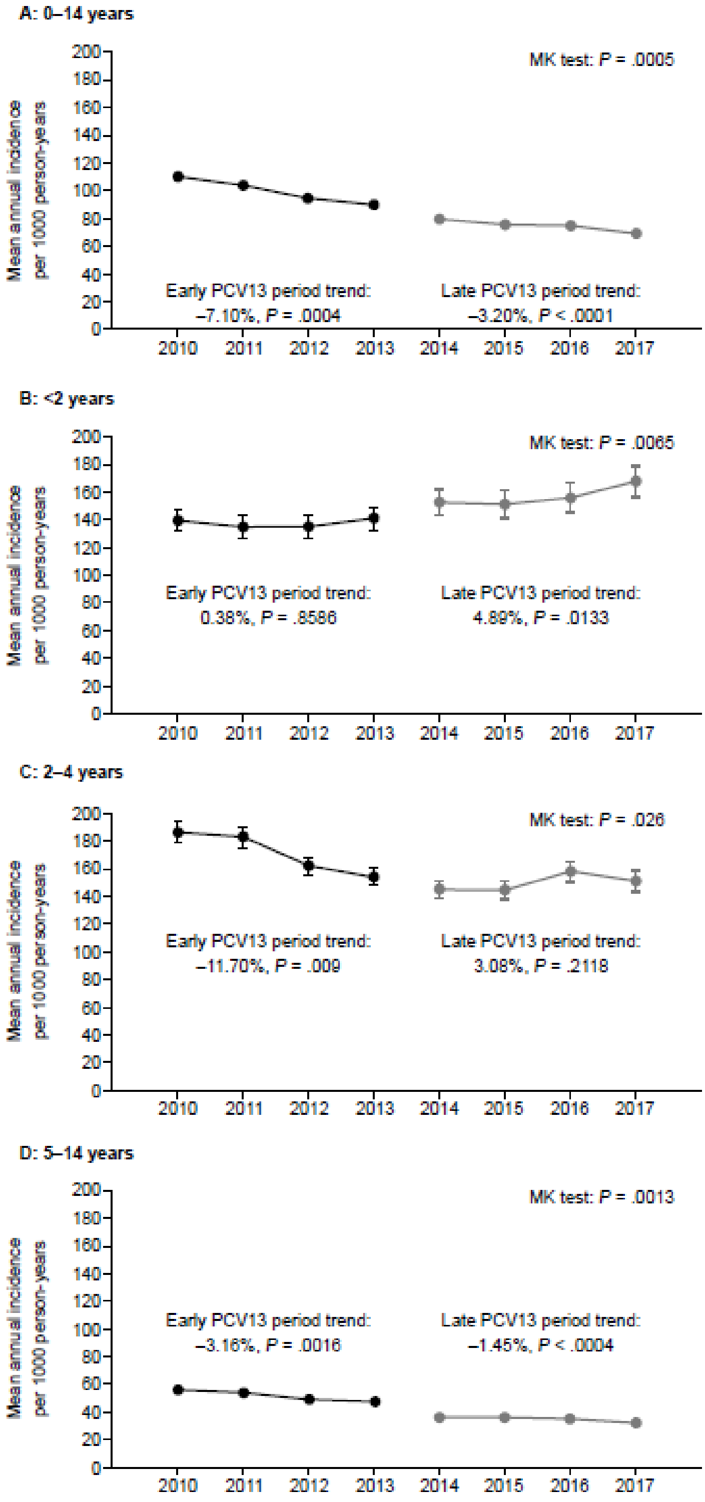

AOM, acute otitis media; MK, Mann–Kendall; PCV13, 13-valent pneumococcal conjugate vaccine.

Supplementary Figure S2. Interrupted time series of mean annual recurrent AOM incidence in the pediatric population: A) 0–14 years; B) <2 years; C) 2–4 years; and D) 5–14 years of age

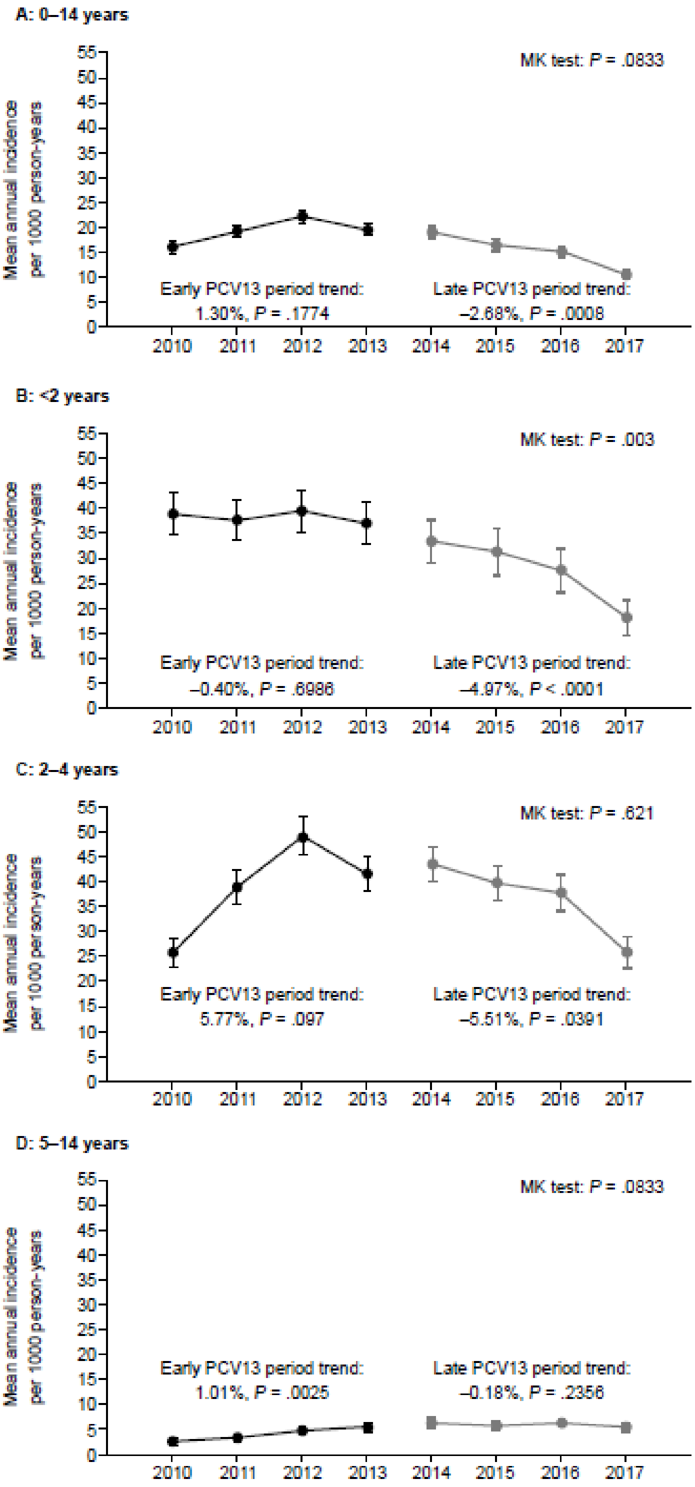

AOM, acute otitis media; MK, Mann–Kendall; PCV13, 13-valent pneumococcal conjugate vaccine.
